# Chemical Characterization of Different Sumac and Pomegranate Extracts Effective against *Botrytis cinerea* Rots

**DOI:** 10.3390/molecules200711941

**Published:** 2015-06-30

**Authors:** Flora V. Romeo, Gabriele Ballistreri, Simona Fabroni, Sonia Pangallo, Maria Giulia Li Destri Nicosia, Leonardo Schena, Paolo Rapisarda

**Affiliations:** 1 Consiglio per la ricerca in agricoltura e l’analisi dell’economia agraria (CRA)-Centro di Ricerca per l’Agrumicoltura e le Colture Mediterranee (CRA-ACM), Corso Savoia 190, Acireale (CT) 95024, Italy; E-Mails: floravaleria.romeo@entecra.it (F.V.R.); gabriele.ballistreri@entecra.it (G.B.); simona.fabroni@entecra.it (S.F.); 2 Dipartimento di Agraria, Università Mediterranea di Reggio Calabria, Località Feo di Vito, Reggio Calabria 89122, Italy; E-Mails: sonia.pangallo@unirc.it (S.P.); giulia.lidestri@unirc.it (M.G.L.D.N.); lschena@unirc.it (L.S.)

**Keywords:** UPLC-PDA-ESI/MS^*n*^, *Punica granatum*, *Rhus coriaria*, anthocyanins, phenols, tannins

## Abstract

Pomegranate (*Punica granatum* L.) peel and sumac (*Rhus coriaria* L.) fruit and leaf extracts were chemically characterized and their ability to inhibit table grape (cv. Italia) rots caused by *Botrytis cinerea* was evaluated on artificially inoculated berries. Different extraction methods were applied and extracts were characterized through Ultra Fast High Performance Liquid Chromatography coupled to Photodiode array detector and Electrospray ionization Mass spectrometer (UPLC-PDA-ESI/MS^*n*^) for their phenol and anthocyanin contents. The concentrated pomegranate peel extract (PGE-C) was the richest in phenols (66.97 g gallic acid equivalents/kg) while the concentrated sumac extract from fruits (SUF-C) showed the highest anthocyanin amount (171.96 mg cyanidin 3-glucoside equivalents/kg). Both phenolic and anthocyanin profile of pomegranate and sumac extracts were quite different: pomegranate extract was rich in cyanidin 3-glucoside, pelargonidin 3-glucoside and ellagic acid derivatives, while sumac extract was characterized by 7-methyl-cyanidin 3-galactoside and gallic acid derivatives. The concentrated extracts from both pomegranate peel and sumac leaves significantly reduced the development of *Botrytis* rots. In particular, the extract from pomegranate peel completely inhibited the pathogen at different intervals of time (0, 12, and 24 h) between treatment and pathogen inoculation on fruits maintained at 22–24 °C and high relative humidity (RH). This extract may represent a valuable alternative to control postharvest fungal rots in view of its high efficacy because of the low cost of pomegranate peel, which is a waste product of processing factories.

## 1. Introduction

Postharvest losses of fruits mainly occur by decay caused by fungal pathogens, which, in addition to the economic damage may produce mycotoxins dangerous to human health [1]. Despite the levels of decay acceptable in postharvest systems are generally below 5%, depending on the commodity and country (developing or industrialized), they can reach levels of up to 50% [2]. Gray mold caused by *Botrytis cinerea*, can cause significant economic losses on many fruits and vegetables and it is generally considered as the most important postharvest pathogen of table grape. The control of fungal postharvest rots is closely linked to the use of pesticides but it is increasingly being restricted throughout the world because of environmental and toxicological problems [3,4]. The continuous use of fungicides, in fact, has faced two major obstacles, increasing public concern regarding contamination of perishables with fungicidal residues, and proliferation of resistance in the pathogen populations. As an example, several strains of *B. cinerea* are resistant to almost all of the commonly used fungicides [5]. The use of SO_2_ generator pads containing sodium metabisulfite is one of the most common methods to control postharvest *B. cinerea* rots on table grapes. However, several reasons, such as its variable efficacy [6], damage caused to berries [7,8] and the allergenic effects recorded in sensitive people restrict SO_2_ application [9,10]. In general, there is an emerging interest to develop “safer” alternative methods for decay control that meet the consumer demand for healthy/organic food products [11].

Natural inhibitors for pathogenic microorganisms have been explored in many plants [12]. Among constituents of plants, polyphenols have received a great deal of attention, in recent years, due to their diverse biological functions. Tannins are high molecular weight phenolic compounds, which are present in many plants, including pomegranate (*Punica granatum* L.) fruit pericarp, whose tannins have remarkable antimicrobial activity [13]. The fruit also contains a rich variety of polyphenols such as anthocyanins, gallotannins, hydroxycinnamic acids derivatives, hydroxybenzoic acids and hydrolysable tannins (as Punicalagin, which is unique to pomegranate and is part of a family of ellagitannins) and gallagyl esters [14]. Approximately 30% of all anthocyanins are concentrated in the peel portion of pomegranate fruit, whose concentration depends on the cultivar type and on the various developmental phases of the fruit [15,16]. Pomegranate has been widely used for several centuries in traditional medicine for a wide variety of diseases including upper respiratory tract infections and influenza [17]. In addition, many investigators have reported that pomegranate peel extracts have a free radical scavenger and potent antioxidant capacity [15,18]. As natural inhibitor for pathogenic microorganisms, the reports about antifungal and antibacterial properties of the pomegranate peel extract are various [1,12,19], depending on the type of the tested microorganism. The above nutraceutical and medical properties are not limited to the edible part of pomegranate fruit, in fact, the non-edible fractions of fruit as well as other plant parts contain even higher amounts of specific nutritionally valuable and biologically active components [14].

Another promising plant source of biologically active molecules is *Rhus coriaria* L., commonly known as sumac, which grows wild in the region extending from Canary Island over the Mediterranean coastline to Iran and Afghanistan. The name derives from “*sumaga*”, meaning red in Syriac [20] and has a long history of use by indigenous peoples for medicinal and other uses [21]. Different parts of sumac possess significant phytochemical components such as tannins, flavonoids, anthocyanins, organic acids, flavones, proteins, fiber, volatile oils, nitrates, and nitrites [22]. The literature well records the potential for useful antimicrobial, antifungal, and antiviral agents from *R. coriaria* extracts [21]. To the best of our knowledge, the sumac extract has not been applied before as an antifungal agent in postharvest control of fruit diseases.

Many publications have documented the antimicrobial activity of natural compounds against different microbial and fungal species [23,24,25,26]. However, there are still few studies about the biological activity of sumac and pomegranate against fungi [1,19,21,27]. In particular, their potential use to control fungal plant pathogens has been rarely investigated [28]. The present study aimed to evaluate different methods to obtain plant extracts from pomegranate peel and sumac fruits and leaves and to assess their efficacy against *B. cinerea* rots on artificially inoculated table grape berries, cv. Italia.

## 2. Results and Discussion

### 2.1. Total Anthocyanins and Total Phenolics

Total anthocyanins and total phenols determined in the extracts (detailed in Table 1) obtained from pomegranate peel, sumac fruits and leaves are shown in Table 2.

**Table 1:** Plant extracts assessed in the present study and relative abbreviations used in the manuscript.

Species	Plant Materials	50% Ethanol/Water Extracts (1:1)	80% Ethanol/Water Extracts (4:1)	Concentrated Aqueous Extract from 80% Ethanol/Water	Water at 40 °C Extracts
Pomegranate	Peels	PGE-50	PGE-80	PGE-C	PGE-H_2_O
Sumac	Fruits	SUF-50	SUF-80	SUF-C	SUF-H_2_O
Sumac	Leaves	SUL-50	SUL-80	SUL-C	SUL-H_2_O

As expected, the aqueous extracts obtained after evaporation of ethanol from 80% ethanol/water extraction (PGE-C, SUF-C and SUL-C) were more concentrated in anthocyanins and phenols than hot water and other hydroalcoholic extracts. Among the concentrated extracts, pomegranate peel (PGE-C) was the richest in phenols and sumac fruit (SUF-C) was the richest in anthocyanins. The water at 40 °C always extracted the lowest quantity of phenols from the different plant materials. For sumac, the leaf extract showed a higher value of phenols than the fruit extract (Table 2). Owing to the high phenol concentration in sumac leaves, many studies confirmed the possibility to use sumac leaves extract in reducing inflammation acting against reactive oxygen species [22,29]. In addition, these extract can be used as tanning agent in the leather industry [22].

**Table 2:** Total anthocyanin and total phenolic contents in pomegranate peel, sumac fruits and leaves extracts.

Extracts ^¥^	Total Anthocyanins ^§^ (mg CGE/kg)	Total Phenolics ^§^ (g GAE/kg)
PGE-C	21.64 ± 0.03 e	66.97 ± 0.67 a
PGE-50	n.q. ^Ω^	9.86 ± 0.20 gf
PGE-80	n.q.	10.36 ± 0.31 f
PGE-H_2_O	n.q.	7.35 ± 0.08 h
SUF-C	171.96 ± 1.51 a	9.47 ± 0.01 g
SUF-50	39.73 ± 0.04 d	5.34 ± 0.43 i
SUF-80	41.97 ± 0.02 c	5.42 ± 0.34 i
SUF-H_2_O	94.92 ± 0.16 b	2.80 ± 0.01 j
SUL-C	-	29.38 ± 0.24 b
SUL-50	-	27.16 ± 0.31 d
SUL-80	-	27.84 ± 0.23 c
SUL-H_2_O	-	15.22 ± 0.13 e

Mean values with different letters (a–j), within the same column are statistically different (*p* < 0.05); ^**¥**^: For abbreviations see Table 1; ^§^: results are expressed as the mean ± standard deviation; ^Ω^: not quantifiable; - not detectable.

In pomegranate extracts, anthocyanins were quantifiable only in the concentrated extract (PGE-C). For the sumac, anthocyanins were found only in fruits while these pigments were not detectable in the leaves. In addition, the amount of anthocyanins in our extracts were higher than that found by Kosar and others [30] who used methanol as extraction solution. The concentrated extract (SUF-C) and the water at 40 °C extract (SUF-H_2_O) showed the highest anthocyanin amounts.

### 2.2. Identification of Anthocyanins by UPLC-PDA-ESI/MS^n^

**Figure 1:**
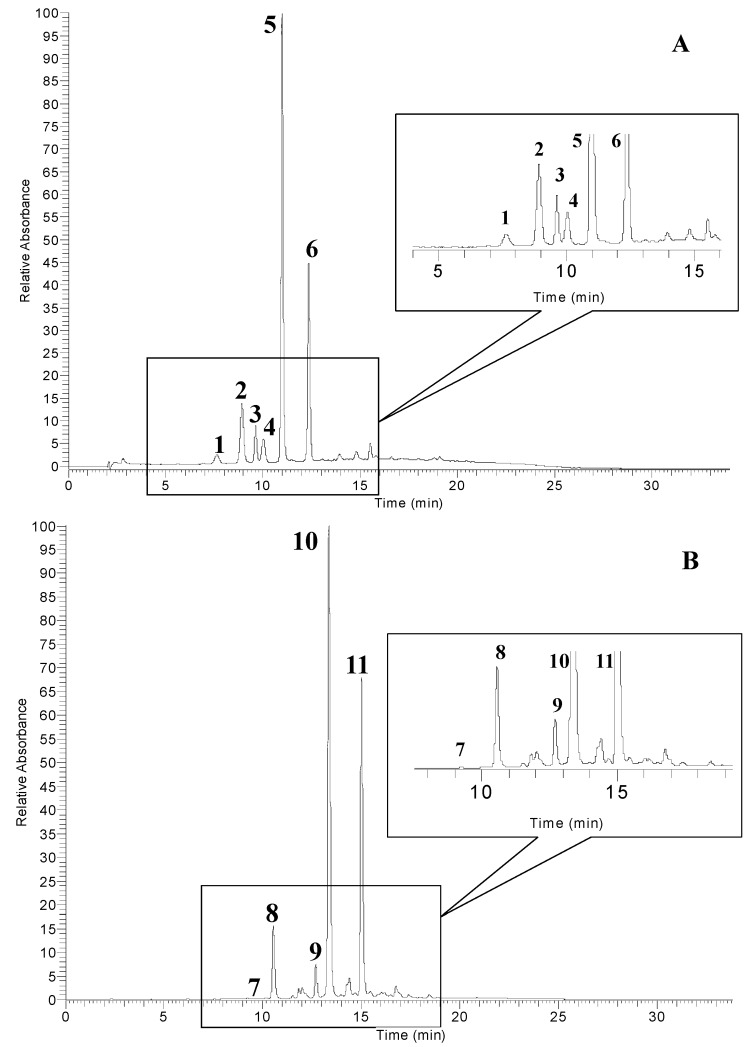
HPLC chromatograms of anthocyanin profiles of pomegranate peel (**A**) and sumac fruit (**B**) detected at 520 nm. Refer to Table 3 for the identification of each numbered peak.

The anthocyanins profile of pomegranate peel extract (PGE-C) is shown in Figure 1A. The anthocyanins, identified on the basis of their retention times (RT) by comparison with authentic standards and by mass spectral analysis, which included MS^1^ and MS^2^ fragmentations, were delphinidin 3,5-diglucoside (peak 1), cyanidin 3,5-diglucoside (peak 2), pelargonidin 3,5-diglucoside (peak 3), delphinidin 3-glucoside (peak 4), cyanidin 3-glucoside (peak 5) and pelargonidin 3-glucoside (peak 6), respectively. Only three anthocyanidins, delphinidin, cyanidin, and pelargonidin, all conjugated with one or two hexose sugars, were found in pomegranate peel extract. This anthocyanidin profile was the same as has previously been found in pomegranate fruit and juice [15,31,32,33]. Classical fragmentation patterns of anthocyanins in ESI positive-mode were recorded and the sequential loss of their saccharide moieties at every induced collision was observed [15,34]. The most abundant anthocyanins in pomegranate peel extract (Table 3) were cyanidin 3-glucoside (49.36%), pelargonidin 3-glucoside (24.62%), and cyanidin 3,5-diglucoside (12.41%); these accounted for more than 85% of the total anthocyanins. The minor anthocyanins were pelargonidin 3,5-diglucoside (5.62%), delphinidin 3-glucoside (4.62%), and delphinidin 3,5-diglucoside (3.37%).

Five anthocyanins were identified in sumac fruit (SUF-C) extract (Figure 1B). After ESI-MS^1^ positive-mode analysis, delphinidin (*m*/*z* 303), cyanidin (*m*/*z* 287), and methylated cyanidin (*m*/*z* 301) structures were identified, while hexose sugars ([M-162]^+^) and galloyl-galactose ([M-314]^+^) losses were recorded. According to their fragmentations and by comparison of the MS data with those reported in the literature [35,36], the identified anthocyanins were delphinidin 3-glucoside (peak 7), cyanidin 3-glucoside (peak 8), cyanidin 3-(2′′-galloyl)galactoside (peak 9), 7-methyl-cyanidin 3-galactoside (peak 10), and 7-methyl-cyanidin 3-(2′′-galloyl)galactoside (peak 11). The fragment ions at *m*/*z* 301 of peak 10 and 11 indicate methyl-cyanidin aglycone yielded after the loss of galloyl-galactoside moiety. An aglycone *m*/*z* of 301 is more commonly encountered for peonidin, but recent studies [35,36] have confirmed the identification of these unusual 7-methylated anthocyanin compounds in sumac fruits by using HPLC-ESI-MS analysis and NMR spectroscopy. The most abundant anthocyanins in sumac fruit extract were 7-methyl-cyanidin 3-galactoside (52.92%), 7-methyl-cyanidin 3-(2′′-galloyl)galactoside (35.14%), and cyanidin 3-glucoside (7.84%), these accounted for more than 95% of the total anthocyanins (Table 3). The minor anthocyanins were cyanidin 3-(2′′-galloyl)galactoside (3.83%), and delphinidin 3-glucoside (0.28%).

**Table 3:** Identification and relative amounts of anthocyanins in concentrated pomegranate peel (PGE-C) and sumac fruit (SUF-C) extracts.

Peak No. ^*a*^	RT (min)	[M]^+^ (*m*/*z*)	MS^n^ (*m*/*z*)	Anthocyanins	Relative Compositions ^*b*^ (%)
Pomegranate peel					
1	7.6	627	465/303	delphinidin 3,5-diglucoside	3.37
2	9.1	611	449/287	cyanidin 3,5-diglucoside	12.41
3	10.0	595	433/271	pelargonidin 3,5-diglucoside	5.62
4	10.3	465	303	delphinidin 3-glucoside	4.62
5	11.0	449	287	cyanidin 3-glucoside	49.36
6	12.6	433	271	pelargonidin 3-glucoside	24.62
Sumac fruit					
7	10.2	465	303	delphinidin 3-glucoside	0.28
8	10.9	449	287	cyanidin 3-glucoside	7.84
9	12.7	601	287	cyanidin 3-(2′′-galloyl) galactoside	3.83
10	13.4	463	301	7-methyl-cyanidin 3-galactoside	52.92
11	15.3	615	301	7-methyl-cyanidin 3-(2′′-galloyl)galactoside	35.14

^*a*^: The numbering is according to Figure 1A,B; ^*b*^: relative content of anthocyanins calculated from peak areas at 520 nm.

### 2.3. Identification of Phenolic Compounds by UPLC-PDA-ESI/MS^n^

**Figure 2:**
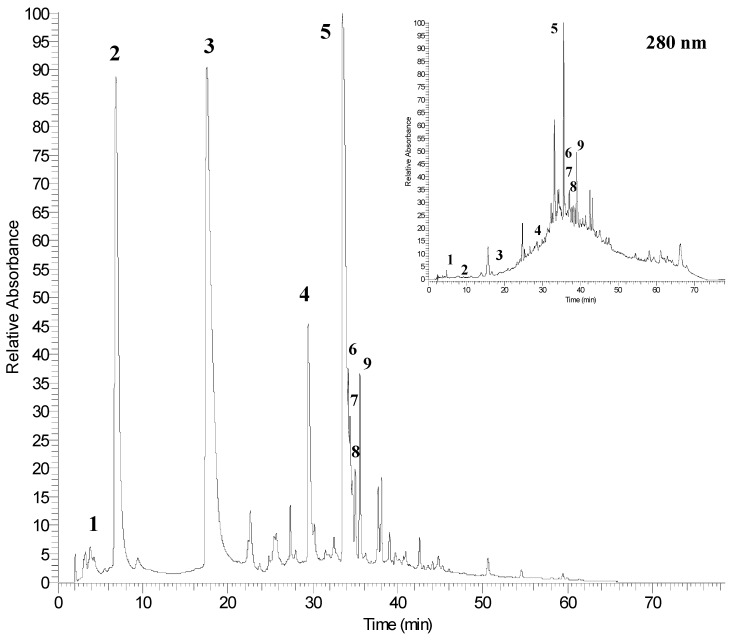
HPLC chromatogram of phenolic compounds of pomegranate peel detected at 378 and 280 nm. Refer to Table 4 for the identification of each numbered peak.

Figure 2 shows the HPLC chromatogram of non-anthocyanins phenolic compounds of pomegranate peel extract (PGE-C) acquired at 378 nm and 280 nm. In this figure, the four major peaks (Table 4) corresponded to punicalagin A and B (peaks 2 and 3), granatin B (peak 4) and ellagic acid (peak 5).

**Table 4:** Identification of phenolic compounds present in concentrated pomegranate peel extract (PGE-C).

Peak No. ^*a*^	RT (min)	[M − H]^−^ (*m*/*z*)	MS^n^ (*m*/*z*)	λ_max_	Phenolic Compounds
1	2.5	169	125	269, 310	gallic acid
2	6.9	1083	781/601	258, 378	punicalagin A
3	17.5	1083	781/601	258, 378	punicalagin B
4	29.4	951	933/613	260, 365	granatin B
5	33.5	301	229/185	254, 367	ellagic acid
6	34.2	-	301	254, 361	ellagic acid derivative
7	34.6	-	301	254, 360	ellagic acid derivative
8	34.9	-	301	255, 362	ellagic acid derivative
9	35.5	-	301	254, 363	ellagic acid derivative

^*a*^: The numbering is according to Figure 2.

The chromatogram acquired at 280 nm revealed the presence of an hydroxybenzoic acid (gallic acid, peak 1) that exhibits a weak absorbance at 378 nm, and other compounds that also absorbs at 378 nm (peaks 6–9) were identified as ellagic acid derivatives. As reported in the literature, only anthocyanins, ellagitannins and gallic acid were detected in pomegranate peel and the major non-anthocyanin phenolic compounds (ellagitannins) of pomegranate products (fruit and juice) are punicalagins [15,32,37]. These compounds were found in the two isomeric forms (peaks 2 and 3), which exhibited a molecular ion at *m*/*z* 1083 and a fragmentation pattern that gave MS^1^ and MS^2^ fragments at *m*/*z* 781 (punicalin) and 601 (gallagic acid), respectively.

Peak 4 exhibited an [M − H]^−^ ion at *m*/*z* 951 that produced fragments at *m*/*z* 933 and 613 in the MS^1^ and MS^2^ experiments, respectively. The fragment at *m*/*z* 933 is typical for castalagin/vescalagin and galloylpunicalin, but these compounds exhibited molecular masses lower than granatin B. The fragment at *m*/*z* 613 derived from the loss of water from the ion at *m*/*z* 951 and from the subsequent losses of ellagic acid and water from the fragment at *m*/*z* 933. Moreover, additional other compounds with ellagitannin structure were peaks 6, 7, 8 and 9, all them sharing the ion at *m*/*z* 301 in their respective MS^*n*^ fragmentation patterns. The ion at *m*/*z* 301 can correspond to both ellagic acid and quercetin. However, ellagic acid produced fragments at *m*/*z* 229 and 185, whereas quercetin generated fragments at *m*/*z* 179 and 151. Furthermore, both compounds differ in their maximum of absorbances (λ_max_) of the respective UV spectra.

**Figure 3:**
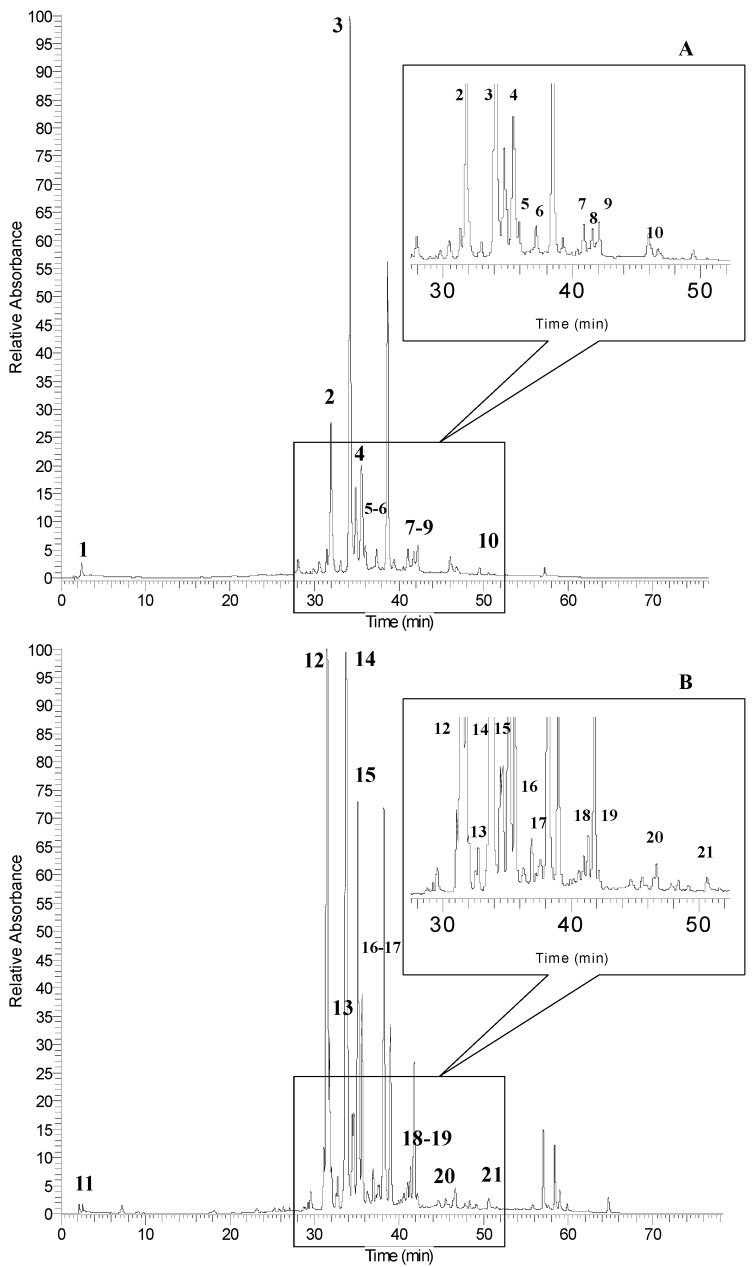
HPLC chromatograms of phenolic compounds of sumac fruit (**A**) and leaves (**B**) detected at 360 nm. Refer to Table 5 for the identification of each numbered peak.

The HPLC chromatograms of non-anthocyanin phenolic profiles of sumac fruit (SUF-C) and leaf (SUL-C) extracts are shown in Figure 3A,B, respectively. As reported in Table 5, ten and eleven phenolic compounds were detected in sumac fruit and leaf extracts, respectively. The first peak was gallic acid at *m*/*z* 169 detected both in sumac fruit and leaves (peak 1 and peak 11). Two flavonoid derivatives (myricetin 3-rhamnoside and quercetin 3-glucoside) were identified both in sumac fruit (peaks 3 and 4) and leaves (peaks 14 and 15).

**Table 5:** Identification of phenolic compounds present in concentrated sumac fruit (SUF-C) and leaf (SUL-C) extracts.

Peak No. ^*a*^	RT (min)	[M − H]^−^ (*m*/*z*)	MS^n^ (*m*/*z*)	λ_max_	Phenolic Compounds
Sumac Fruit					
1	2.6	169	125	269, 310	gallic acid
2	31.9	-	301	255, 354	quercetin derivative
3	33.9	463	316	257, 366	myricetin 3-rhamnoside (see Figure 4A)
4	35.3	463	301	255, 351	quercetin 3-glucoside (see Figure 4B)
5	37.3	939	921/787/169	280	pentagalloyl-glucoside
6	39.9	1091	939/169	282	hexagalloyl-glucoside
7	41.9	1243	1091/169	281	heptagalloyl-glucoside
8	43.5	1395	1243/169	285	octagalloyl-glucoside
9	45.5	1547	1395/169	283	nonagalloyl-glucoside
10	50.5	1699	1547/169	278	decagalloyl-glucoside
Sumac leaves					
11	2.5	169	125	269, 310	gallic acid
12	31.4	-	316	255, 366	myricetin derivative
13	31.8	-	301	255, 346	quercetin derivative
14	33.9	463	316	255, 366	myricetin 3-rhamnoside (see Figure 4A)
15	35.3	463	301	255, 352	quercetin 3-glucoside (see Figure 4B)
16	37.1	939	921/787/169	280	pentagalloyl-glucoside
17	39.7	1091	939/169	280	hexagalloyl-glucoside
18	41.7	1243	1091/169	281	heptagalloyl-glucoside
19	43.2	1395	1243/169	285	octagalloyl-glucoside
20	45.4	1547	1395/169	284	nonagalloyl-glucoside
21	50.6	1699	1547/169	277	decagalloyl-glucoside

^*a*^: The numbering is according to Figure 3A,B.

**Figure 4:**
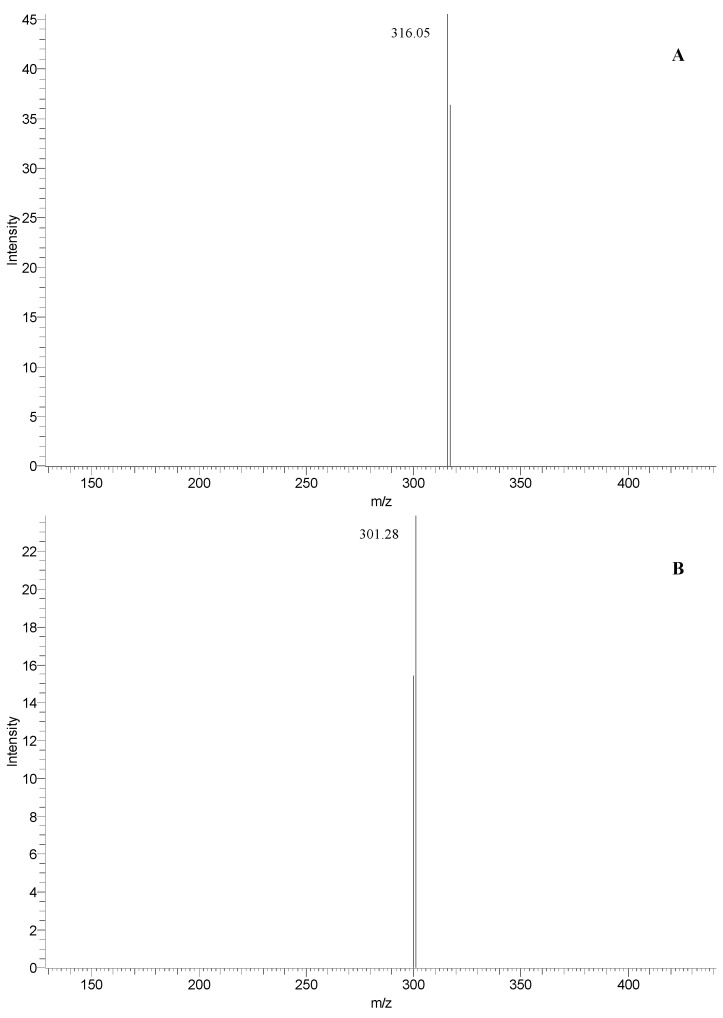
Fragmentation pathways of the ions at *m*/*z* 463 at RT 33.9 and 35.3 min, with the assignment to myricetin 3-rhamnoside (**A**) and quercetin 3-glucoside (**B**), respectively.

These compounds exhibited the same [M − H]^−^ ions at *m*/*z* 463 but a different fragmentation pathways. The MS^1^ spectrum of the peaks at RT 33.9 min (Figure 4A) shows the fragment ion at *m*/*z* 613 indicative of the myricetin, while the peaks at RT 35.3 min gave a fragment at *m*/*z* 301 (Figure 4B), corresponding to the deprotonated quercetin. Based on this evidence and the previous literature data [35,38], as well as by direct comparison with standards using UV spectra and co-chromatography in several solvent systems, we can assess the simultaneous presence of myricetin 3-rhamnoside and quercetin 3-glucoside both in fruits and leaves of *Rhus coriaria* L. Moreover, the presence of other quercetin and myricetin derivatives was highlighted (peak 2 and peaks 12 and 13 for sumac fruit and leaves, respectively).

The main tannin compounds present in the *Rhus* family are gallotannins [30]. As reported in Table 5, sumac fruit and leaves showed the same gallotannin composition from penta to decagalloyl-glucoside. These compounds were separated by a constant mass value of 152 Da corresponding to the molecular weight of the galloyl moiety. As confirmed by previous studies [38], the loss of galloyl residue attached to the pentagalloyl-glucose core via *m*-depside bonds is favored compared to the loss of gallic acid (170 Da) directly linked to the glucose core, due to the higher activation energy needed to break this type of bond. For this reason, the ion relative to the loss of gallic acid at *m*/*z* 921 only in the pentagalloyl-glucoside fragmentation pathway was highlighted. This type of fragmentation is typical of galloylated compounds with up to five galloyl units [38,39].

### 2.4. Efficacy of Sumac and Pomegranate Extracts against Botrytis cinerea on Artificially Inoculated Grape Berries

**Figure 5:**
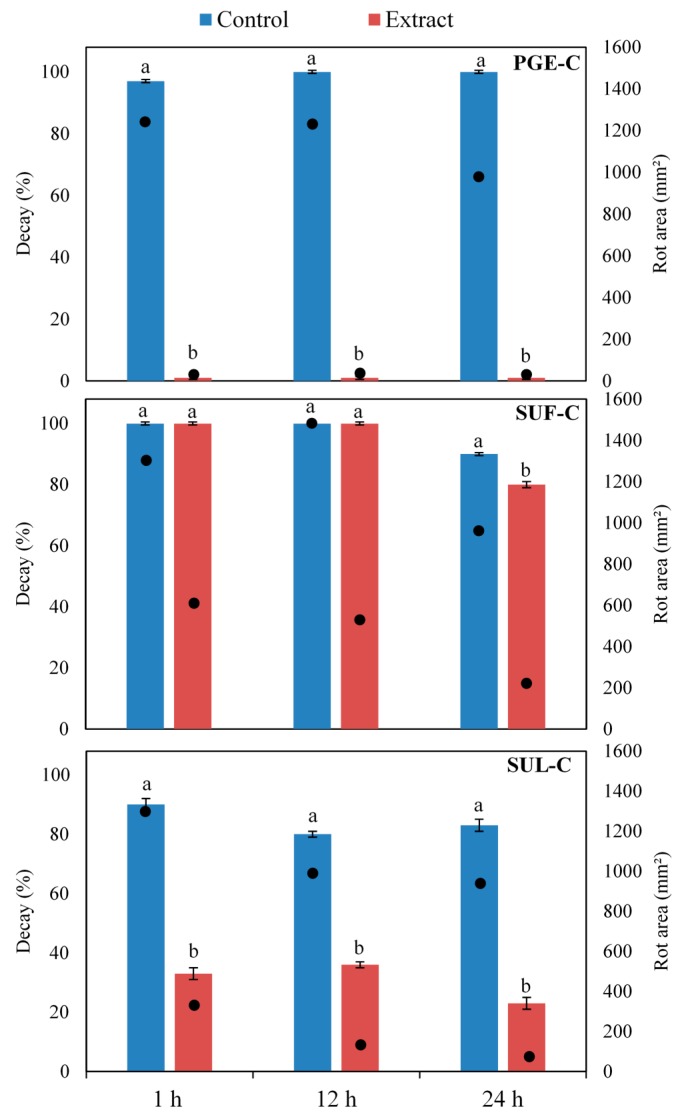
Incidence of decays (**columns**) and extension of rots (**black dots**) on table grape berries treated with concentrated extracts of pomegranate peel (PGE-C), sumac fruits (SUF-C), or sumac leaves (SUL-C) and artificially inoculated *Botrytis cinerea* after 1, 12 or 24 h. Wounds mock treated with a solution of 1% citric acid and inoculated with the pathogen, served as controls. For each extract and assessment time (1, 12 and 24 h), values not sharing common letters are statistically different according to Duncan’s multiple range test (*p* ≤ 0.05). Standard error bars are shown on each column.

Among tested extracts, the concentrated ones (PGE-C, SUL-C and SUF-C) were able to reduce the incidence and/or the extension of *B. cinerea* rots (Figure 5). On the contrary, other extracts (PGE-H_2_O, SUF-H_2_O, SUL-H_2_0, PGE-80, PGE-50, SUF-80, SUF-50, SUL-80 and SUL-50) did not significantly reduce rots or had a limited inhibition effect (data not shown). These results are in agreement with the chemical composition since total phenols reached the highest content in concentrated extracts (Table 2). A direct correlation between total phenols and antimicrobial activity is well documented [12]. Phenols may also play an important role by inducing resistance in the host and/or by reducing the production of mycotoxins [40,41]. These results highlight the importance of the extraction method, which is strictly related to the type of solvent and the solvent ratio and strongly affects the phenolic yield and the consequent biological activity [14].

Among concentrated extracts, pomegranate peel (PGE-C) was the most effective completely inhibiting the development of *B. cinerea* in all the tested time intervals (Figure 5). As regard to sumac, extracts from fruits were overall less performing as compared to those from leaves. On fruits treated with SUL-C, the rotted areas on artificially inoculated berries was reduced by 70%, 81% and 92% as compared to the positive control at 1, 12 and 24 h, respectively (Figure 5). Similarly, the incidence of infected fruit was significantly reduced by 63%, 55% and 72% in the three assessment times, respectively. On the contrary, SUF-C determined a significant reduction of the incidence of rots only when applied 24 before the pathogen, although a reduction of the area of rots was revealed in all times (Figure 5).

The observed differences in biological activity among PGE-C, SUL-C and SUF-C extracts may be related to their qualitative and quantitative composition. In particular, the concentration of total phenolics was very high in PGE-C, intermediate in SUL-C and low in SUF-C (Table 2). Moreover, pomegranate and sumac extracts exhibited different tannin patterns (Table 4 and Table 5). Among ellagitannins, punicalagins, which are the main compounds in the peels, are the most bioactive compounds having antifungal activity [1].

## 3. Experimental Section

### 3.1. Pomegranate and Sumac Extracts

Extracts obtained from pomegranate (*Punica granatum* L.) peels and from sumac (*Rhus coriaria* L.) leaves and fruits were investigated in the present study. Pomegranate fruits cv. “Mollar de Elche”, at an optimum stage of ripening were from an orchard located in Acireale (Italy). Sumac fruits and leaves were collected in Buccheri (Siracusa, Italy), a typical production area of this crop. After harvesting, all fruits and leaves were immediately transferred to the CRA-ACM laboratories (Acireale, Italy). Then, all plant portions were cut into pieces and oven dried at 40 °C up to constant weight, and then powdered in a homegrinder (Mx Type A505, Moulinex, Ecully Cedex, France). The dried powder of sumac fruits was extracted for 8 h with *n*-hexane (VWR Chemicals, Milan, Italy) to remove lipid components using a Soxhlet apparatus. The defatted sumac fruits were put in an oven at 50 °C for 8 h to remove the residue of *n*-hexane. Afterwards, the powder was extracted with the extracting solutions. All extracts were obtained from grinded tissues with water or water/ethanol (95%, food grade, Fichera, S. Venerina, Italy) solutions with a ratio of 1:10 (*w*/*v*). From both pomegranate and sumac plant materials, four kind of hydroalcoholic extracts were tested: (1) 50% ethanol/water (1:1) (*v*/*v*); (2) 80% ethanol/water (4:1); (3) a concentrated aqueous extract obtained from 80% ethanol/water mixture after evaporation of ethanol by using a rotary evaporator (Rotavapor RE111, Büchi, Cornaredo, Italy) under vacuum at 40 °C; and finally; (4) a water extract obtained using distilled water at 40 °C (Table 1). The extracting solutions were supplemented with 1% citric acid (Sigma-Aldrich, Milan, Italy) except for the solution used for the preparation of concentrated extracts (80% ethanol/water (4:1)). For this latter extract a 0.2% citric acid solution was utilized in order to have in the final extract (after the evaporation of ethanol) a concentration of approximately 1%. The solutions were left in an orbital shaker (711D model, Tecnolab, Messina, Italy) for 24 h. Then, the solutions were filtered through Miracloth paper (Calbiochem, Vimodrone, Italy) and through a 0.45 μm membrane filter (Albet, Barcelona, Spain) for the subsequent analyses.

### 3.2. Determination of Total Anthocyanins and Total Phenolic Content of the Plant Extracts

The total anthocyanins in the extracts were assayed by the pH differential method [42]. The absorbance values of appropriately diluted extracts were measured at 520 nm by a UV-Vis spectrophotometer (Varian Cary 100 Scan, Palo Alto, CA, USA). The total anthocyanins was expressed as mg of cyanidin 3-glucoside equivalents (CGE)/kg of extract. The total phenolic content in the extracts was determined according to the Folin–Ciocalteu (FC) colorimetric method [43]. The extracts were mixed with 5 mL of FC commercial reagent (previously diluted with water, 1:10 *v*/*v*) and 4 mL of a 7.5% sodium carbonate solution. The mixture was stirred for 2 h at room temperature away from strong light. The absorbance of the resulting blue solution was measured spectrophotometrically at 765 nm and the total phenolic content was expressed as g of gallic acid equivalents (GAE)/kg of extract.

### 3.3. UPLC-PDA-ESI/MS^n^ Analyses

For identification of anthocyanins, PGE-C and SUF-C extracts were loaded onto a C18 Bond Elut SPE cartridges (Varian Inc., Palo Alto, CA, USA) pre-washed in methanol and then pre-equilibrated in water. Anthocyanins were adsorbed onto the C18 Bond Elut column, while other soluble compounds were removed by washing the cartridges with water. Then anthocyanins were eluted with acidified methanol (containing 1% of formic acid). The acidified methanol solutions were evaporated to dryness, and then the dry fractions were re-dissolved in a 7% formic acid aqueous solution. Samples were filtered through a 0.45 μm membrane filter (Albet, Barcelona, Spain) and injected into the UPLC-MS^*n*^ chromatographic system (see below) for identification of individual anthocyanins. The standards of cyanidin 3-glucoside, cyanidin 3,5-diglucoside, delphinidin 3-glucoside, delphinidin 3,5-diglucoside, pelargonidin 3-glucoside, pelargonidin 3,5-diglucoside, and myricetin 3-rhamnoside were purchased from Extrasynthese (Genay, France). Gallic acid, quercetin 3-glucoside, ellagic acid, and punicalagin were purchased from (Sigma-Aldrich). All other chemicals were of analytical grade (Sigma-Aldrich). Solvents for chromatography were HPLC grade (Merck KGaA, Darmstadt, Germany).

For identification of non-anthocyanins phenolic compounds, a sample of PGE-C, SUF-C and SUL-C extracts was diluted in mobile phase solvent A (water containing 0.3% of formic acid), filtered through a 0.45 μm filter, and injected directly into the column.

Separations of anthocyanins and non-anthocyanins phenolic compounds were conducted on an Onyx Monolithic C18 column (100 × 3.0 mm i.d., monolithic particle size; Phenomenex, Torrance, CA, USA), using an Ultra Fast HPLC system coupled to a photodiode array (PDA) detector and a Finnigan LXQ ion trap equipped with an electrospray ionization (ESI) interface in a series configuration (Thermo Electron, San Jose, CA, USA). Two different binary gradients were used. A binary gradient composed of water containing 7% of formic acid (solvent A) and methanol (solvent B) was used for the separation of anthocyanins. The gradient was run as follows: from 5% to 40% of B in 20 min, isocratic for 7 min, followed by re-equilibrating the column to initial conditions. A binary gradient composed of water containing 0.3% formic acid (solvent A) and acetonitrile containing 0.3% formic acid (solvent B) was used for the separation of phenolic compounds. The gradient was run as follows: 0 min, 5% B, 10 min, 2% B, 50 min, 28% B, 60 min, 43% B, isocratic for 20 min, followed by re-equilibrating the column to initial conditions. The flow rate was 300 μL/min, the column temperature was maintained at 30 °C and the injection volume was 20 μL, both for anthocyanins and phenolics analysis. Chromatograms were recorded at 280, 320, 378 and 520 nm.

Operating parameters of the mass spectrometer were the same for both anthocyanins and phenolics analysis: spray voltage 5.00 kV, capillary temperature 275 °C, capillary voltage 19 V. A sheath gas flow rate of 30 arb (arbitrary units) was applied, and the auxiliary and sweep gas were set at 15 and 4 arb, respectively. Preliminary positive and negative tunings were carried out with direct injection of diluted solutions of cyanidin 3-glucoside and punicalagin, respectively at a flow rate of 5 μL/min and the voltages of the optical lenses were optimized by using TunePlus Xcalibur v. 2.0.7 software (Thermo Electron, San Jose, CA, USA). MS full-scan acquisition was carried out from *m*/*z* 100 to 2000, in both positive and negative mode. Then, chosen peaks were isolated in the ion trap and fragmented by an MS^*n*^ scan acquisition. The MS^*n*^ spectra were obtained by using a collision energy of 13%–17% of instrument maximum, operating with an isolation width (*m*/*z*) of 1.5. The mass spectrometry data were acquired in the positive ionization mode for anthocyanins and in the negative ionization mode for other phenolic compounds.

The anthocyanins and phenolics were identified by using their retention times (*t*_R_), UV-Vis spectra, co-chromatography with standard in several solvent systems, MS and MS^*n*^ spectral data operating in positive and negative ion mode, respectively. In addition, comparison of the MS data with those of pure standards and/or those reported in literature was performed. The relative composition (%) of the individual anthocyanins and phenolics were calculated from the peak areas of the chromatograms detected at 520 and 320 nm, respectively, using Xcalibur v. 2.0.7 software (Thermo Electron).

### 3.4. Preparation of the Pathogen Inoculum

A pure culture of *B. cinerea* was obtained from infected grape berries. Conidia were directly collected from decayed fruits, serially diluted with sterile water and plated on potato dextrose agar (Sigma-Aldrich) in order to obtain single conidia colonies. Pure cultures were kept on PDA at 5 °C for long-term storage or grown at 22 °C for 7–10 days to produce inocula. Conidia were collected with a spatula, suspended in sterile distilled water, filtered through a double layer of sterile muslin cloth (Artsana, Rome, Italy), vortexed for 1 min to assure uniform mixing, and diluted to the final concentration of 2 × 10^6^ conidia mL^−1^. A hemocytometer chamber (Brand Gmbh COKG, Wertheim, Germany) was utilized to evaluate the concentration of conidia.

### 3.5. In-Vivo Assays of Extracts on Artificially Inoculated Grape Berries

Tests were performed with all extracts (Table 1) on table grape berries, cv. Italia, of uniform size and ripeness from an organic farm located in Calabria (Southern Italy). Individual berries were surface sterilized by immersion in a 2% sodium hypochlorite solution for 1 min, washed twice with tap water, air-dried and glued on polypropylene honeycomb panels (Nidaplast, Thiant, France) using a double-sided tape. Berries were kept 1 cm apart to avoid nesting and wounded with a nail in the equatorial zone.

Wounds were treated by applying 10 μL of extracts and inoculated with an equal volume of a conidia suspension of *B. cinerea* after 1, 12 or 24 h. Wounds treated with 1% citric acid and inoculated with the pathogen, served as a control. Fruits glued on polypropylene honeycomb panels were always maintained at room temperature (22–24 °C) in lidded plastic boxes containing wet paper to ensure high relative humidity (RH). The incidence of infected wounds and the area of rots (with and without mycelia) were evaluated after 4 days of incubation. The formula of the ellipse using the two perpendicular axes as input data was utilized to determine the rotted area. Each treatment was applied on 30 grape berries arranged in three groups of 10 berries.

### 3.6. Statistical Analysis

Data analysis was carried out with the SPSS software (version 21.0, IBM Statistics, Armonk, NY, USA). One-way ANOVA analysis of variance was used to test the effect of the different variables on the measured factors. Duncan’s test was used to perform multiple comparisons between the means (*p* ≤ 0.05). The data of decay % converted into Bliss angular values (arcsin √%) were submitted to one-way ANOVA analysis and compared using Duncan’s test (*p* ≤ 0.05).

## 4. Conclusions

Although results of the present study may be considered preliminary, they suggest PGE-C and SUL-C as potential effective plant extracts to control *B. cinerea* rots as well as other postharvest fungal pathogens. An important feature is the high efficacy of PGE-C, which completely inhibited *B. cinerea* unlike the great majority of currently available alternative control means, which only determine a delay in the development of rots. Furthermore, this extract proved to also be every effective when applied 12 and 24 h before the pathogen, indicating that its biological activity lasts for several hours after its application on the infection site. From a practical point of view, this result suggests the possible use of the extract just before or soon after harvest to protect fruits during critical phases such as harvesting and packaging, in which fungal pathogens can penetrate trough wounds and establish infections. Finally, the wide availability and the low cost of pomegranate peel, which is a waste product of the processing factories, and of the sumac plant that grows wild in the Mediterranean area, may facilitate the development of new commercial formulations.
